# Regionality and Temporal Dynamics of Sequestration and Relocation of Cardenolides in the Monarch Butterfly, *Danaus plexippus*

**DOI:** 10.1007/s10886-025-01572-8

**Published:** 2025-02-04

**Authors:** Anja Betz, Birgit Höglinger, Frank Walker, Georg Petschenka

**Affiliations:** 1 Department of Applied Entomology, University of Hohenheim, Stuttgart, Germany; 2 KomBioTa—Center for Biodiversity and Integrative Taxonomy, University of Hohenheim and State Museum of Natural History, Stuttgart, Germany

**Keywords:** Isolated gut, Cardenolide sequestration, Selectivity, Epithelial transport, *Danaus plexippus*, Cardenolide polarity

## Abstract

The adaptation of monarch butterflies (*Danaus plexippus*) to milkweed plants and their ability to sequester toxic cardenolides is a model system for plant-herbivore coevolution. However, the physiological mechanisms underlying cardenolide sequestration and its temporal dynamics remain largely unknown. Here, we show that the polar cardenolide ouabain passes through the isolated midgut epithelium of *D. plexippus in vitro* and is also absorbed into the body cavity of monarch caterpillars. Remarkably, the same pattern was observed in caterpillars of the related, but non-sequestering milkweed butterfly *Euploea core*, and even in the non-adapted Solanaceae specialist *Manduca sexta*, although uptake across gut epithelia occurred at a lower rate. Furthermore, we demonstrated that cardenolides begin to cross the epithelium in the anterior part of the intestine and can be detected in body tissues as soon as one minute after ingestion. Finally, we show that not all cardenolides are translocated into butterfly tissues during metamorphosis, and that the most apolar cardenolides are removed with the last caterpillar exuviae. As a result, adult butterflies contain no cardenolides less polar than the milkweed cardenolide calactin. We conclude that uptake by the intestinal epithelium is a very rapid process and that quantitative differences in cardenolide sequestration among lepidopteran caterpillars are only partially mediated by the gut epithelium, likely involving additional mechanisms such as metabolism or excretion. In addition, the translocation of cardenolides from the caterpillar is a selective process which may be due to the limited mobility of highly apolar cardenolides.

**Supplementary Information:**

The online version contains supplementary material available at 10.1007/s10886-025-01572-8.

## Introduction

Sequestration in insects is defined as ‘the selective uptake, transport, modification, storage and deployment of plant secondary chemicals for the insect’s own defense’ (Heckel 2018). The ability to sequester plant metabolites is found in species from at least six insect orders (Duffey 1980; Nishida 2002; Opitz and Müller 2009). In the Lepidoptera, sequestration occurs in many species and includes the storage of different chemical classes such as terpenic, phenolic and nitrogen-containing metabolites (Nishida 2002; Opitz and Müller 2009). Cardenolides, which belong to the terpenic metabolites, have been extensively studied in chemical ecology (Brower and Moffitt 1974; Rothschild et al. 1975; Nishida 2002). These toxic steroids have been identified in over 12 plant families (Dobler et al. 2015), in which they occur in all plant tissues (Agrawal et al. 2012).

The toxicity of cardenolides in animals is caused by inhibition of an essential cation carrier, the Na^+^/K^+^ -ATPase (Vaughan and Jungreis 1977; Holzinger and Wink 1996; Dobler et al. 2015). Cardenolide sequestering insects express resistant Na^+^/ K^+^ -ATPases, which was first observed in the monarch butterfly (*Danaus plexippus*; Nymphalidae: Danaini) (Vaughan and Jungreis 1977; Holzinger et al. 1992). In monarch butterflies and many other sequestering Lepidoptera, defense toxins can only be obtained during larval development (Beran and Petschenka 2022). In addition, the amount of toxins stored, depends on the type of chemical compound and the species of host plant (Nishida 2002; Opitz and Müller 2009).

During the process of sequestration, ingested molecules have to pass several barriers to reach the body tissues where the toxins are stored. In Lepidoptera and many other insects, the peritrophic membrane (PM), which surrounds the food bolus and separates the luminal compartment of the midgut from the epithelial cells (Fiandra et al. 2009), is the first barrier after ingestion of plant material. After passing through the PM, molecules must cross the wall of the gut, which consists of a monolayer of epithelial cells (Wolfersberger et al. 1986). In general, molecules can pass through the gut epithelium via either paracellular (between cells) or transcellular (through cells) pathways (Huang et al. 2015). While the paracellular pathway likely is regulated by septate junctions (SJs) maintaining a diffusion barrier, the transcellular pathway may be controlled by ATP-consuming efflux carriers such as ABC-transporters (Petschenka et al. 2013; Dobler et al. 2015; Huang et al. 2015).

How sequestered toxins are transported across the gut epithelium is largely unknown. The underlying mechanisms have only been elucidated in a few systems, such as the cytochrome P450-mediated transport of nicotine in the midgut of *Manduca sexta* caterpillars (Kumar et al. 2014). For cardenolides, carrier-mediated uptake has been proposed in monarch caterpillars and the large milkweed bug (*Oncopeltus fasciatus*) (Detzel and Wink 1995; Frick and Wink 1995), but the nature of these carriers has never been established. Other studies have demonstrated the presence of cardenolides in the midgut epithelium of *D. plexippus* and *O. fasciatus*, and suggested that cardenolide transport occurs across the midgut epithelium possibly by diffusion (Yoder et al. 1976; Scudder and Meredith 1982; Dreisbach et al. 2023). Among other characteristics, the polarity of a compound determines its transport, and it is thought that apolar cardenolides cross the gut epithelium passively, whereas polar ones may require active transport (Agrawal et al. 2012). Furthermore, sequestering insects have been found to convert apolar cardenolides to more polar forms for storage (Nishio 1980; Seiber et al. 1980; Martin et al. 1992; Agrawal et al. 2012).

In this study, we investigated the sequestration of cardenolides in caterpillars of the monarch butterfly (*D. plexippus*), the related, cardenolide-consuming but non-sequestering common crow butterfly (*Euploea core*) (Malcolm and Rothschild 1983; Petschenka and Agrawal 2015; Dreisbach et al. 2023), and the tobacco hornworm (*Manduca sexta*), a Solanaceae specialist, which is not adapted to cardenolides. We focused on cardenolide transport across the isolated intestinal midgut epithelium as well as on sequestration in the whole caterpillar. Initially, we determined the time-dependent uptake of a mixture of the water-soluble model cardenolide ouabain and the milkweed cardenolide uzarin across isolated midguts of *D. plexippus.* Using this approach, we tested the hypothesis that ouabain can permeate the midgut faster than the similarly polar but larger uzarin. In addition, we tested the hypothesis that ouabain can permeate the midgut of *D. plexippus*, whereas the midguts of *E. core* and *M. sexta* are impermeable to cardenolides, as the latter two species do not sequester cardenolides. Therefore, we compared the uptake of ouabain in isolated midguts and sequestration in whole caterpillars of all three species. We assumed that ouabain would cross the epithelium and be sequestered into the body tissues of *D. plexippus*, whereas it would not be taken up in the bodies of *E. core* and *M. sexta.* In addition, we investigated the temporal dynamics of the sequestration of ouabain and the non-polar cardenolide digitoxin in *D. plexippus* caterpillars using feeding assays. Because both ouabain and digitoxin are cardenolides not found in their natural diet, milkweed (*Asclepias* spp.), we additionally studied the time course and regionality of sequestration of milkweed cardenolides in monarch caterpillars and evaluated cardenolide relocation during metamorphosis.

## Materials and Methods

### Cultivation of Plants

To induce seed germination, *Asclepias* seeds were nicked with a scalpel blade, embedded in moist tissue, placed in Petri dishes and sealed with breathable tape. The dishes were then kept in the dark at 28 °C for 4 days. After germination, seedlings were planted in trays and transplanted two weeks later into 11 × 11 × 12 cm pots. Plants were kept in the greenhouse at 23–28/21–24 °C day/night, 16/8 h light/dark, fertilized weekly with a 0.2% dilution (v/v) of universal fertilizer (Wuxal Super N: P: K 8: 8: 6, Hauert MANNA Düngerwerke GmBH, Nürnberg, Germany) and watered as needed.

### Butterfly Rearing

Rearing of *D. plexippus* (origin: Portugal) and *E. core* (origin: Southeast Asia) was done under greenhouse conditions (see above). The caterpillars of *D. plexippus* and *E. core* used for the experiments were obtained from long-term colonies that had been maintained over multiple generations. The adult *D. plexippus* butterflies were kept in a 2 × 1 × 2 m flight cage and *E. core* butterflies in a 2 × 2 × 2 m flight cage. Both species were fed with a 10% sucrose solution and nectar plants, including *A. curassavica*, *Lantana camara*, and *Pentas lanceolata*. Additionally, crushed seeds of *Heliotropium indicum* and *H. foertherianum* were provided to *E. core* butterflies as a source of pheromone precursors. To maintain humidity, a fogging system (Micro Rain Systems, Altenburg, Germany) was used to spray the butterflies with water for 2 min, eight times a day. Caterpillars of *D. plexippus* were either reared on *A. curassavica* or *Asclepias syriaca* depending on the experimental requirements (see below). Caterpillars of *E. core* were reared on *A. curassavica*. Caterpillars of the tobacco hornworm (*Manduca sexta*) reared on tobacco plants (*Nicotiana tabacum*) and additional plants for experiments were kindly provided by the Department of Molecular Botany at the University of Hohenheim (Stuttgart, Germany).

### Experiments

#### In Vitro Transfer of Ouabain and Uzarin through Isolated Midguts

**Fig. 1:**
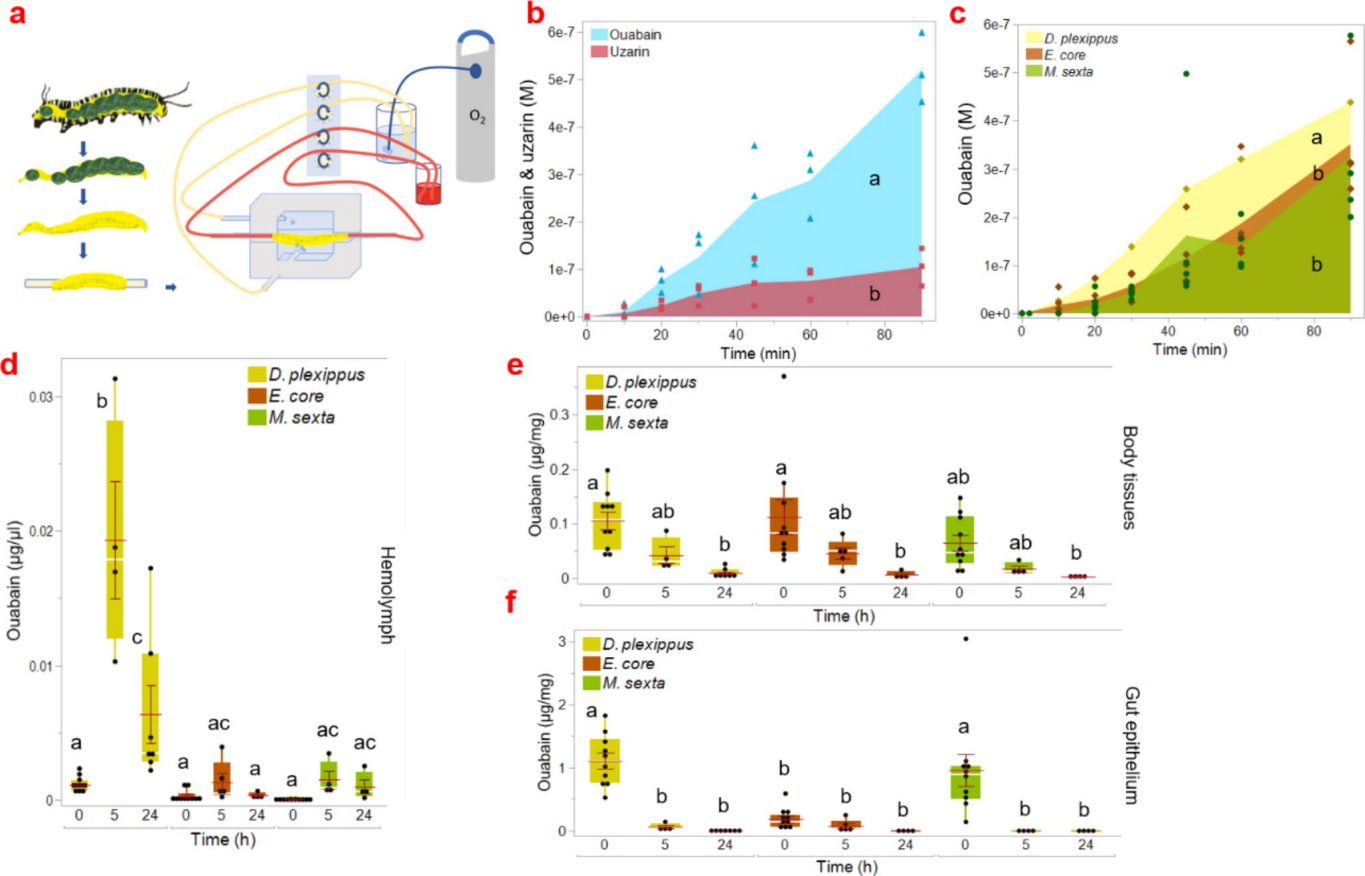
Sequestration of polar cardenolides across isolated midguts and in whole caterpillars. Permeability of the midgut epithelium was studied by placing the isolated midguts of caterpillars in a perfusion chamber (**a**). A mixture of ouabain and uzarin was tested in isolated midguts of *D. plexippus* (**b**). In addition, time dependent ouabain uptake was measured in isolated midguts of *D. plexippus*,* E. core* and *M. sexta.* Symbols show individual data points, and edges of colored areas connect the means of the raw data (**c**). In addition, we tested ouabain uptake in the whole caterpillar. Assuming complete extraction, ouabain concentrations were determined by HPLC-DAD using calibration curves in all three species in the hemolymph (**d**), body tissues (**e**) and the gut epithelium (**f**). Note that 0 h indicates samples taken after 90 min of continuous ouabain access. No further ouabain was administered up to 5 and 24 h. Boxes indicate interquartile ranges and whiskers indicate maximum and minimum values, white lines indicate medians, and red lines show means. Black dots represent single data points, error bars indicate SE. Different letters indicate significant differences between treatments

We used isolated caterpillar midguts to evaluate transepithelial cardenolide permeation in monarch caterpillars. Following the same approach, we tested if midguts of the cardenolide-feeding but non-sequestering *E. core* and of the non-adapted *M. sexta* are impermeable to the polar cardenolide ouabain. Caterpillars of *D. plexippus*, *E. core* (both last instar), and *M. sexta* (4th instar, but similar in size) were cooled at -20 °C for 6 min, decapitated and sectioned dorsally from caudal to cranial while immersed in ice-cold phosphate-buffered saline (PBS, pH 7.4). The midgut was isolated and the PM including all plant content was pulled out. Afterwards, the empty midgut was carefully slipped over a perforated piece of ismaprene tubing (inside: 1.02 mm ⌀, outside 2.7 mm ⌀; ParMed, Ismatec). Next the tube was placed in a custom-made permeation chamber (made of acrylic glass; experimental setup: Fig. 1a), filled with artificial hemolymph (5 mM K_2_SO_4_, 4.8 mM MgSO_4_, 1 mM CaCl_2_, 10 mM K citrate, 10 mM l-alanine, 10 mM l-glutamine, 10 mM glucose, 190 mM sucrose, 5 mM Tris; pH 7 adjusted with 1 N HCl) (Casartelli et al. 2007). The chamber was then carefully closed while the midgut preparation was clamped in the front and back of the chamber. The hemolymphatic solution (10 ml) was pumped from a beaker (20 ml) through the chamber and back to the beaker via a peristaltic pump (Reglo ICC, Ismatec) using ismaprene tubing (inside: 1.02 mm ⌀, outside 2.7 mm ⌀; ParMed, Ismatec) at a constant flow rate of 0.4 ml/min and oxygenated with an oxygen cylinder using a silicone tube (inside: 4 mm ⌀, Air Liquide Medical GmbH, Düsseldorf, Germany) for 5 min every 15 min. The perforated ismaprene tube (where the midgut was slipped on) was connected to additional ismaprene tubing (inside: 1.02 mm ⌀, outside 2.7 mm ⌀; ParMed, Ismatec) and perfused with a luminal buffer (same buffer as hemolymphatic solution but adjusted to pH 8.3 with 1 N HCl) at a flow rate of 0.05 ml/min. The total volume of the luminal buffer was 1.5 ml and we used a 15 ml Falcon tube as a reservoir. The luminal buffer contained either a mixture of 10 mM ouabain (Carl Roth GmbH, Karlsruhe, Germany), 5 mM of the milkweed cardenolide uzarin (Pytolab GmbH & Co. KG, Vestenbergsgreuth, Germany) and 1 mg/ml amaranth (Waldeck GmbH & Co KG, Münster, Germany) (*D. plexippusn* = 3) or 10 mM ouabain and 1 mg/ml amaranth (*E. core n = 5*,* M. sexta*,* n = 6*,* D. plexippus n = 1*). Amaranth is a dye that cannot pass through cell membranes (Dow 1981) and therefore was included as a control for leakage.

We collected 1 ml samples from the hemolymphatic solution at 0, 10, 20, 30, 45, 60, and 90 min in 2 ml screw cap micro tubes and immediately replaced the volume with fresh artificial hemolymph. After 90 min, the midgut was removed from the chamber and tissue viability was assessed after incubation with 200 µl of trypan blue (undiluted, Sigma-Aldrich, USA) for 5 min in a Petri dish at room temperature (i.e. only dead cells are stained blue by the dye, only samples from vital tissue were taken into analysis). The intestinal tissue was then washed three times with PBS and observed under a stereomicroscope (VWR International, USA). Buffer samples collected during the experiment were frozen at -80 °C and lyophilized (Alpha 2–4 LDplus, Martin Christ, Osterode, Germany). Dried samples were dissolved in 500 µl MeOH/H_2_O (1:1) and filtered (nylon syringe filter 0.45 μm, Ø 13 mm, Carl Roth GmbH & Co.KG, Karlsruhe, Germany) into HPLC vials. Ouabain and uzarin were quantified by high performance liquid chromatography-mass spectrometry (HPLC-MS; see below). Potential amaranth contamination was assessed using an ultra-high performance liquid chromatography (U-HPLC) system connected to a diode array detector (DAD; see below) and samples were discarded if amaranth was detected.

To compare the results obtained for ouabain transport across the isolated midgut epithelium, we additionally studied the uptake of ouabain in whole caterpillars. Caterpillars of each species at the same stages as described above (*D. plexippusn* = 21, *E. coren* = 18, *M. sextan* = 18) were fed with ouabain-coated leaf discs (40 µl 10 mM in MeOH; let to evaporate before feeding). Coated leaf discs of *A. curassavica* were used for *E. core* and *D. plexippus*, and coated leaf discs of *N. tabacum* were used for *M. sexta*. Caterpillars were fed ad libitum for 90 min on the ouabain-coated discs (note that caterpillars typically feed intermittently, see Fig. S1 for numbers of leaf discs consumed per caterpillar). A subset of caterpillars was then placed on untreated *A. curassavica* (*D. plexippus*,* E. core*) or *N. tabacum* (*M. sexta*) leaves and allowed to feed for additional 5–24 h. After 90 min of continuous access to ouabain, as well as after 5–24 h of further feeding without access to ouabain, a hemolymph sample (10 µl) was collected with a glass capillary after cutting off one of the anterior tentacles of *D. plexippus* and *E. core*. For *M. sexta*, the dorsocaudal horn was cut off. Then, caterpillars were frozen at -20 °C for later dissection. For chemical analysis, hemolymph samples were extracted as described below and caterpillars were dissected into gut epithelia (PM and dietary content removed) and remaining body tissues (i.e. the caterpillar integument plus the adhering tissues) and extracted as described below. Detection of ouabain was carried out by HPLC-MS (see below). To compare the sequestration of a relatively polar and a relatively unpolar cardenolide in whole monarch caterpillars, we also examined the time course of the sequestration of ouabain (polar) and digitoxin (more unpolar), which are not present in the natural host plants of *D. plexippus* (experimental setup Fig. S2a). In contrast to previous work (Frick and Wink 1995), we examined sequestration also at earlier time points (see supplementary material for details on methods).

#### Regionality and Time-Dependent Sequestration in *D. plexippus*

The aim of this experiment was to determine how quickly natural cardenolides obtained from *A. curassavica* pass from the gut lumen into the hemocoel. In addition, we wanted to test whether the sequestration of cardenolides is restricted to certain regions of the midgut. To this end, we followed the passage of ingested milkweed through the gut and measured the sequestration of cardenolides in the corresponding portion of the gut epithelium and the surrounding body tissues. We reared *D. plexippus* caterpillars on *A. syriaca* plants (low cardenolide content) until the last larval instar (L5). To replace the green leaf material in the gut, we fed caterpillars Hokkaido pumpkin pulp, which can be easily distinguished from milkweed leaf material by its orange color. After 24 h, we placed the caterpillars on detached leaves of *A. curassavica*, which has a higher cardenolide content and produces structurally different cardenolides compared to *A. syriaca*, allowing chromatographic differentiation between the cardenolides from the two plant species. After the start of feeding, caterpillars were removed from *A. curassavica* leaves at 1, 5, 10, 30, 60 and 90 min, a hemolymph sample (10 µl) was collected as described above (*n* = 4 per timepoint) and caterpillars were frozen at -20 °C for later dissection. As a control, caterpillars were taken directly from the pumpkin diet without being placed on *A. curassavica*.

For dissection, the caterpillars were opened dorsally after thawing and the gut and body tissues were separated correspondingly to how far the green plant material had travelled through the gut. Only gut portions filled with *A. curassavica* leaf material and the corresponding portion of adjacent body tissues were subjected to cardenolide analysis. The food bolus was removed from the gut tissue by pulling out the PM including the dietary content. Samples were frozen at -80 °C, freeze-dried, weighed on a microbalance (0.001 g; Cubis II, Sartorius Corporate Administration GmbH, Göttingen, Germany), extracted and analyzed as described below. Because the cardenolide concentrations in the hemolymph measured by HPLC-DAD were very low, we additionally quantified the concentrations of calotropin and calactin, two of the most abundant cardenolides sequestered by the monarch caterpillar when feeding *on A. curassavica* (Rosenthal and Berenbaum 1992), by HPLC-MS (see below).

#### Cardenolide Relocation During Metamorphosis

To evaluate the distribution of cardenolides during metamorphosis, we extracted caterpillar tissues at different stages of chrysalis development and from newly emerged adult butterflies. Stages were differentiated as follows: (I) freshly attached caterpillars in the so-called J-hang-position (Salisbury and Salisbury 2000); (II) J-hang just before shedding the last caterpillar exuvia; (III) soft pupae; and (IV) freshly emerged butterflies (*n* = 6 per stage). Tissues were separated into cuticle (can be easily removed during J-hang stages, see supplementary for details S3), body tissues (excluding wings), wings and gut (including dietary content), frozen at -80 °C, freeze-dried, weighed on a microbalance, extracted, and analyzed with HPLC-DAD as described below.

#### Extraction of Caterpillar Tissues and Butterfly Guts

Samples were placed in 2 ml screw cap micro tubes containing 0.9 g of zirconia beads (2.3 mm, Carl Roth GmbH, Karlsruhe, Germany) and 1 ml methanol was added. Next, samples were homogenized twice with a FastPrep-24 homogenizer (MP Biomedicals, Eschwege, Germany) for 45 s (6.5 m/s). After 3 min of centrifugation (16,000 g at 22 °C; 5417R, Eppendorf, Hamburg, Germany), we transferred the supernatants into new 2 ml screw cap micro tubes. Again, 1 ml of methanol was added and the procedure was repeated. Subsequently, pooled supernatants were evaporated to dryness in a vacuum centrifuge (RVC 2–25 CDplus, Martin Christ, Osterode, Germany). Finally, the dry residues were dissolved in methanol for HPLC-DAD or in methanol/water 1:1 for HPLC-MS analysis (hemolymph samples in 100 µl; body tissues and gut epithelium samples in 200 µl, additionally diluted 1:10 for HPLC-MS). Samples were homogenized (45 s; 6.5 m/s), centrifuged (3 min, 16,000 g, 22 °C) and filtered through nylon syringe filters (0.45 μm, Ø 13 mm, Carl Roth GmbH & Co.KG, Karlsruhe, Germany) into HPLC vials.

#### Extraction of Butterfly Wings and Body Tissues

Freeze-dried samples were collected in a 15 ml centrifuge tube containing two ceramic beads (6.35 mm, MP Biomedicals, Graffenstaden, France) and 2 ml of methanol. Samples were homogenized twice using the FastPrep-24 homogenizer as described above. After centrifugation for 10 min (1000 g at 22 °C), the supernatants were transferred to glass test tubes. Sample extraction was repeated with 2 ml methanol. Pooled supernatants (4 ml) were evaporated to dryness in a vacuum centrifuge. The dried residue was dissolved in 500 µl of methanol and transferred to 2 ml screw cap micro tubes. This procedure was repeated twice using an ultrasonic bath during the last round to completely dissolve any residues. The remaining 1.5 ml of each sample was evaporated to dryness again. Finally, the samples were dissolved in 200 µl methanol, homogenized (45 s; 6.5 m/s), centrifuged (3 min, 16,000 g, 22 °C), and filtered (nylon syringe filter 0.45 μm, Ø 13 mm) into HPLC vials.

#### Quantification of Ouabain, Uzarin, Digitoxin, Calotropin and Calactin by HPLC-MS

We quantified the cardenolide content of the sample extracts using a LTQ Velos HPLC-MS system (Thermo Scientific, USA) equipped with an Accela 1250 pump and an Accela AS autosampler. Cardenolides were separated by reversed-phase chromatography on a XB 150/3 Kinetex C18, 2.6 μm column (Phenomenex, Aschaffenburg, Germany) maintained at 40 °C. Three microliters of sample extract were injected and eluted at a constant flow rate of 0.5 ml/min using an increasing 5% acetonitrile in water/acetonitrile gradient as follows: 0–1.1 min 5% acetonitrile; 1.1–15 min 5–90% acetonitrile; 15–17 min 90% acetonitrile; 17–17.1 min 90–95% acetonitrile; 17.1–20 min 95% acetonitrile.

The ion trap mass spectrometer was operated in negative electrospray ionization mode for all cardenolides mentioned above with m/z ranges 100–2000, S-Lens RF level: -92 V, resolution of 0.1 at m/z 300 as follows: ouabain: m/z 583 and m/z 629 (M + 2Na), uzarin: m/z 697 and m/z 743 (M + 2Na), digitoxin: m/z 763 and 809 Na (M + 2Na), calactin/calotropin: m/z 577 (M + 2Na). The following mass spectrometer settings were used: spray voltage (3 kV), conversion dynode (-14.99 kV), voltages settings of multiplier 1/2 (-1372 V, -1420 V), capillary temperature 300 °C, source heater temperature 300 °C; sheath-, auxiliary- and sweep gas 30, 15, 1 x AU, respectively. The HPLC-MS data of the cardenolides were analyzed using Xcalibur software version 2.2 SP1.84 (ThermoFisher Scientific). Mass-specific quantification was determined using purified standards of the toxin of interest in a calibration curve (0.01, 0.1, 0.5, and 2.5 mg/ml).

#### Quantification of Amaranth by U-HPLC-DAD

Amaranth was quantified on a Dionex Ultimate 3000 HPLC system (Thermo Scientific, USA) using a 150/3 Waters ACCLAIMTM C30, 3 μm column (Thermo scientific, Germany). Three microliters of sample extract were injected. Amaranth was eluted at a constant flow rate of 0.5 ml/min using an increasing formate buffer (pH 3.7 + 5% acetonitrile) / acetonitrile gradient as follows: 0–1 min 5% acetonitrile; 1–11 min 5–95% acetonitrile; 11–13 min 90% acetonitrile; 13–13.1 min 90 − 5% acetonitrile; 13.1–16 min 5% acetonitrile. Peaks were detected with a DAD (Dionex Ultimate 3000, Thermo Scientific) at 520 nm. Amaranth content in samples was determined using an external amaranth calibration curve at 0.5, 1 and 5 mg/L. Chromatograms were analyzed using the software Chromeleon 7, version 7.2 SR5.

#### Quantification of Cardenolides by HPLC-DAD

Cardenolides were quantified on an Agilent Infinity 1260 HPLC system (Agilent Technologies, USA) using an EC 150/4.6 NUCLEODUR C18 Gravity, 3 μm column (Macherey-Nagel, Düren, Germany) maintained at 30 °C. The injection volume of the sample was 15 µl. Cardenolides were eluted at a constant flow rate of 0.7 ml/min using an increasing water/acetonitrile gradient as follows: 0–2 min 16% acetonitrile, 2–25 min 16–70% acetonitrile, 25–30 min 70–95% acetonitrile, 30–35 min 95% acetonitrile, 35–37 min 95 − 16% acetonitrile, 10 min reconditioning at 16% acetonitrile. Peaks were detected with a DAD at 218 ± 4 nm (absorbance spectra 200–400 nm). Peaks showing a characteristic symmetric absorption spectrum with a maximum between 218 and 222 nm were classified as cardenolides (Malcolm and Zalucki 1996). The cardenolide content of the samples was determined using a digitoxin calibration curve at 5, 10, 25 50, 100, 250, 500, 750, and 1000 µg/ml.

#### Data Analysis

Statistical analysis was performed using R 1.4.1103 (R Foundation). Probability values < 0.05 were considered statistically significant. Raw data were tested for within group normality using a *Shapiro Wilk test*. For normally distributed data, a linear model was fitted, while when within-group normality was rejected, a generalized linear model using the Gaussian family for continuous data was fitted.

Ouabain and uzarin uptake was analyzed with a linear model (lm(toxin content ~ toxin + time). Since ouabain uptake did not appear to be affected by uzarin, all data on ouabain uptake (i.e., from the ouabain + uzarin experiment, Fig. 1a, *n* = 3 and from the ouabain only experiment, Fig. 1b, *n* = 1) were combined for statistical analysis. The ouabain uptake data (in vitro and in vivo) were not normally distributed within groups, so a GLM with a Gaussian family was performed (glm(ouabain content ~ species + time + time: species, family="gaussian”). Since the interaction of ouabain level and time was not significant, the model was reduced (glm(ouabain ~ species + time, family="gaussian”). This was followed by an *ANOVA* followed by a comparison of means with a *Tukey post hoc test*. We followed the same procedure for the additional experiment on digitoxin and ouabain uptake in vivo in *D. plexippus*, without reducing the model because the interaction was significant (glm(cardenolide content ~ toxin + time + time: toxin, family="gaussian”). A GLM (glm(cardenolide concentration ~ stage, family="gaussian”)) was performed for cardenolide concentration in tissues during metamorphosis for wing, body, and gut data. Cuticle data were analyzed by LM (lm(cardenolide concentration ~ stage)). No statistics were possible for *A. curassavica* cardenolide uptake because the data set contained many zero values and was highly scattered. The quantified cardenolides refer only to those that are sequestered from *A. curassavica* plants.

For the comparisons of overall cardenolide polarities between samples, we calculated the polarity index according to Rasmann and Agrawal (2011). Briefly, the mean polarity index (P) of a sample was determined by the sum of the retention time (RT) of each cardenolide peak multiplied by its peak area (Pi) divided by the total area of all cardenolide peaks in the sample (Rasmann and Agrawal 2011). The graphical representation of the data was done using JMP Pro 17.

## Results

To study the midgut epithelium as a barrier for sequestered cardenolides, we constructed an in vitro permeation chamber and tested the permeation of ouabain and the slightly less polar milkweed cardenolide uzarin in *D. plexippus*. We found that the amount of ouabain, which has a lower molecular weight (ouabain = 584.65 g/mol; uzarin = 698.79 g/mol), that crossed the isolated midgut epithelium was significantly higher than that of uzarin (F_1,29_ = 26.02; *P* < 0.001; Fig. 1b). In addition, we compared the absorption of ouabain across the isolated midgut epithelia of *D. plexippus*, *E. core*, and *M. sexta* caterpillars (Fig. 1c). Contrary to our expectations, we found that ouabain was also able to cross the isolated midguts of all three species (χ^2^_2_ = 20.94, *P* < 0.001). Our results showed that the midgut of *M. sexta* and *E. core* did not differ in permeability, while the *D. plexippus* midgut was slightly more permeable to ouabain (*E. core & M. sexta* vs. *D. plexippus*, *P* < 0.001 each; *E. core vs. M. sexta*, *P* = 0.9). Following this discovery, we tested if ouabain would also penetrate the gut in vivo when ingested by caterpillars. Ouabain was found in the hemolymph of all three species, although at different concentrations. The highest concentration was detected in *D. plexippus*, while there was no significant difference between the other two species (χ^2^_2_ = 17.42, *P* < 0.001; *E. core & M. sexta* vs. *D. plexippus*, *P* < 0.001 each; *E. core vs. M. sexta*, *P* = 0.99). Five hours after an initial period of 90 min of ouabain feeding, hemolymph ouabain concentrations had increased and subsequently decreased after 24 h. This overall pattern was similar for all species, but only in *D. plexippus* were the differences between timepoints significant (*D. plexippus 0 h* vs. *D. plexippus 5 h*, *P* < 0.001; *D. plexippus 0 h* vs. *D. plexippus 24 h*, *P* < 0.01, *D. plexippus 5 h* vs. *D. plexippus 24 h*, *P* < 0.001). Furthermore, we found different ouabain concentrations in the body tissues over time in all species (χ^2^_8_ = 35.011, *P* < 0.001), but no difference in ouabain concentrations immediately after 90 min of continuous ouabain supply (i.e. timepoint “*0 h*” in our graphs; *E. core 0 & M. sexta 0 h* vs. *D. plexippus 0 h*, *P* = 1, *P* = 0.7). After 5 and 24 h after ouabain feeding, there was no difference in ouabain concentrations in body tissues between the species, and a decrease over this time period was observed in all species (Fig. 1e). In the gut epithelial tissue, 90 min after continuous feeding, no differences in the ouabain concentrations were found in *D. plexippus* and *M. sexta* (*P* = 0.99), but ouabain concentrations in *E. core* were lower (*M. sexta & D. plexippus 0 h vs. E. core 0 h*, *P* < 0.001 each; Fig. 1f).

Furthermore, we examined the time course of the sequestration of ouabain (polar) and digitoxin (more unpolar), which are not present in the natural host plants of *D. plexippus* (see supplementary material) but are often used as model cardenolides in related studies. In contrast to previous work (Frick and Wink 1995), we examined sequestration at earlier time points. Specifically, we tested whether the two cardenolides with different polarities were sequestered at different rates. Remarkably, both ouabain and digitoxin were detected in the gut epithelia and body tissues of all caterpillars (*n* = 4) as early as 5 min after the start of feeding. We found that digitoxin was sequestered at a higher rate in body tissues (χ^2^_6_ = 66.22, *P* < 0.001) and gut epithelium (χ^2^_6_ = 23.14, *P* < 0.001) than ouabain. However, the occurrence of both cardenolides in the hemolymph was less consistent, but after 30 min (digitoxin) or 90 min (ouabain) hemolymph samples from all caterpillars consistently contained digitoxin and ouabain. Digitoxin was found in higher concentrations than ouabain (data from 90 to 180 min, F_1,15_ = 6.04, *P* < 0.02, Supplementary S2).

**Fig. 2:**
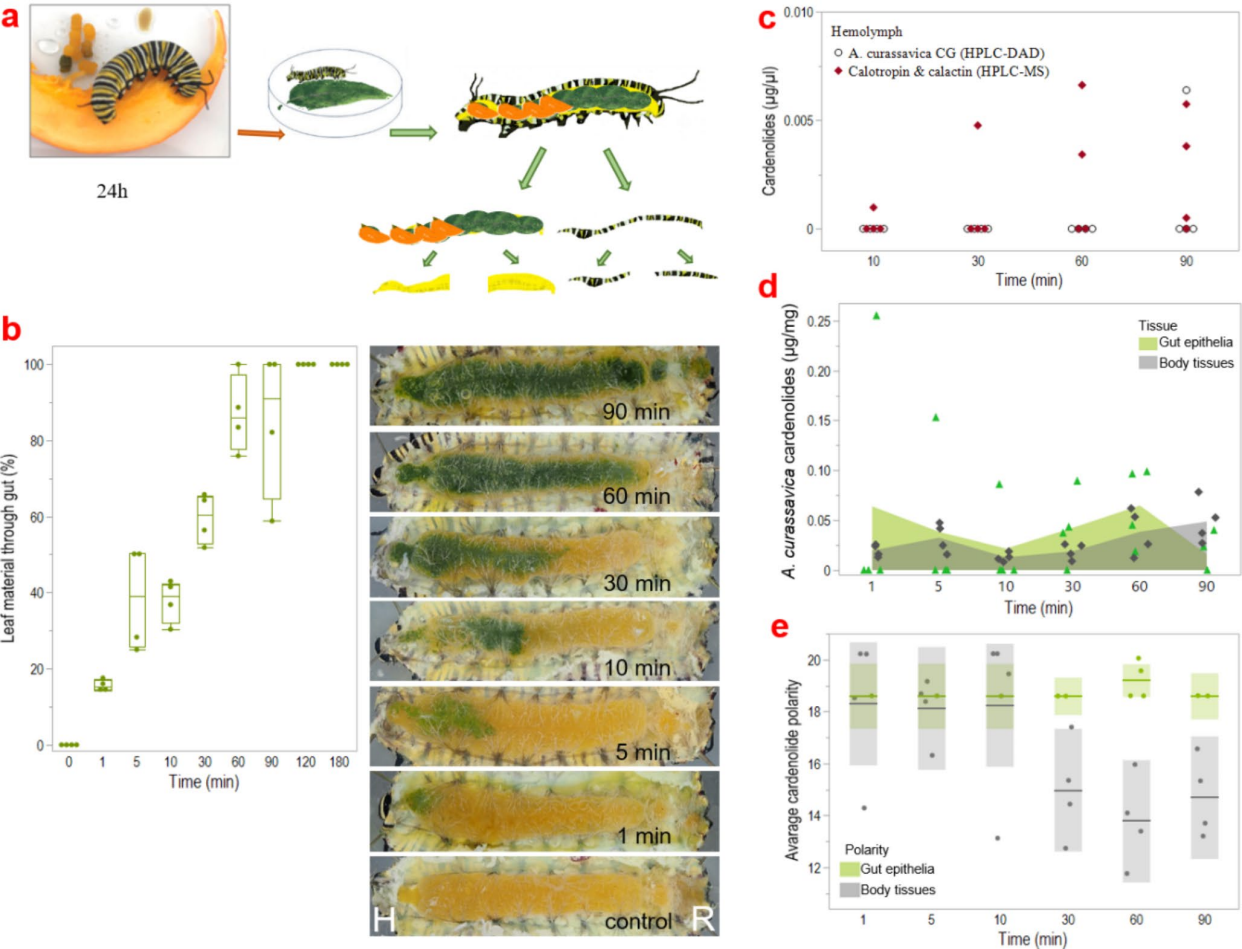
Time course and regionality of cardenolide sequestration from milkweed in *D. plexippus*. To monitor the passage of ingested milkweed leaves, we fed the caterpillars pumpkin pulp before feeding them milkweed leaves (**a**). By dissecting the caterpillars at different times (orange parts indicate pumpkin, green parts leaf material), we were able to determine how far the *A. curassavica* leaf material had traveled through the gut. The corresponding pictures oriented head “H” left and rear end “R” right show dissected caterpillars after 0, 1, 5, 10, 30, 60, and 90 min feeding on *A. curassavica* leaves (**b**). In parallel, we quantified the cardenolides derived from *A. curassavica* (assuming complete extraction) in the hemolymph of the caterpillars at the corresponding time points by HPLC-DAD using calibration curves. The dominant *A. curassavica* cardenolides calotropin and calactin (red diamonds), were quantified using HPLC-MS. Total *A. curassavica* cardenolides (including calactin and calotropin) were assessed by HPLC-DAD using a digitoxin calibration curve (black circles, note lower sensitivity) (**c**). The concentration of cardenolides found in the epithelium of the gut portion filled with leaves and the corresponding region of body tissues was measured as a function of time after feeding. Edges of areas represent the mean value of the data, individual data points representing cardenolide concentrations in the gut epithelia are shown as green triangles, while those of the body tissues are shown as black diamonds (**d**). In addition, the polarity index of the detected cardenolides was calculated, each dot represents one sample (note that *A. curassavica* cardenolides were not found in every sample of caterpillar tissue), and the lines show mean values (**e**)

In addition to the sequestration assays with the non-milkweed cardenolides ouabain and digitoxin, we investigated the time course and regionality of sequestration using the natural host plant *A. curassavica* in a separate feeding experiment with last instar caterpillars of *D. plexippus*. Using pumpkin-fed caterpillars (Fig. 2a), we were able to determine how far the *A. curassavica* leaf material had traveled through the gut based on the color difference. We found that a gut passage of *A. curassavica* leaf material in *D. plexippus* caterpillars was completed after 90–120 min (i.e., the pumpkin pulp was completely replaced by leaf material; Fig. 2b). In addition to the gut epithelium and body tissues, we also sampled the hemolymph before dissecting the caterpillars. We found *A. curassavica* cardenolides (i.e., calotropin and calactin) in one caterpillar as early as 10 min after the start of feeding using HPLC-MS, but the first cardenolides were not detected until 90 min using the less sensitive HPLC-DAD (Fig. 2c). When we compared the polarity patterns between the cardenolides detected in the gut epithelium and the corresponding body tissues, we found an increasing polarity of cardenolides in the body tissues compared to the gut epithelium over time. However, at most time points, the gut epithelial samples analyzed using HPLC-DAD only contained a single *A. curassavica* cardenolide with a RT of 18.6 min (Fig. 2d). Remarkably, we found that cardenolides were already present in the body tissues of all caterpillars 1 min after feeding (Fig. 2e). Relating these results to the distance traveled by leaf material in the gut, we found that cardenolides first cross the gut barrier after about 15% passage (i.e., once the leaf material reaches the midgut).

**Fig. 3:**
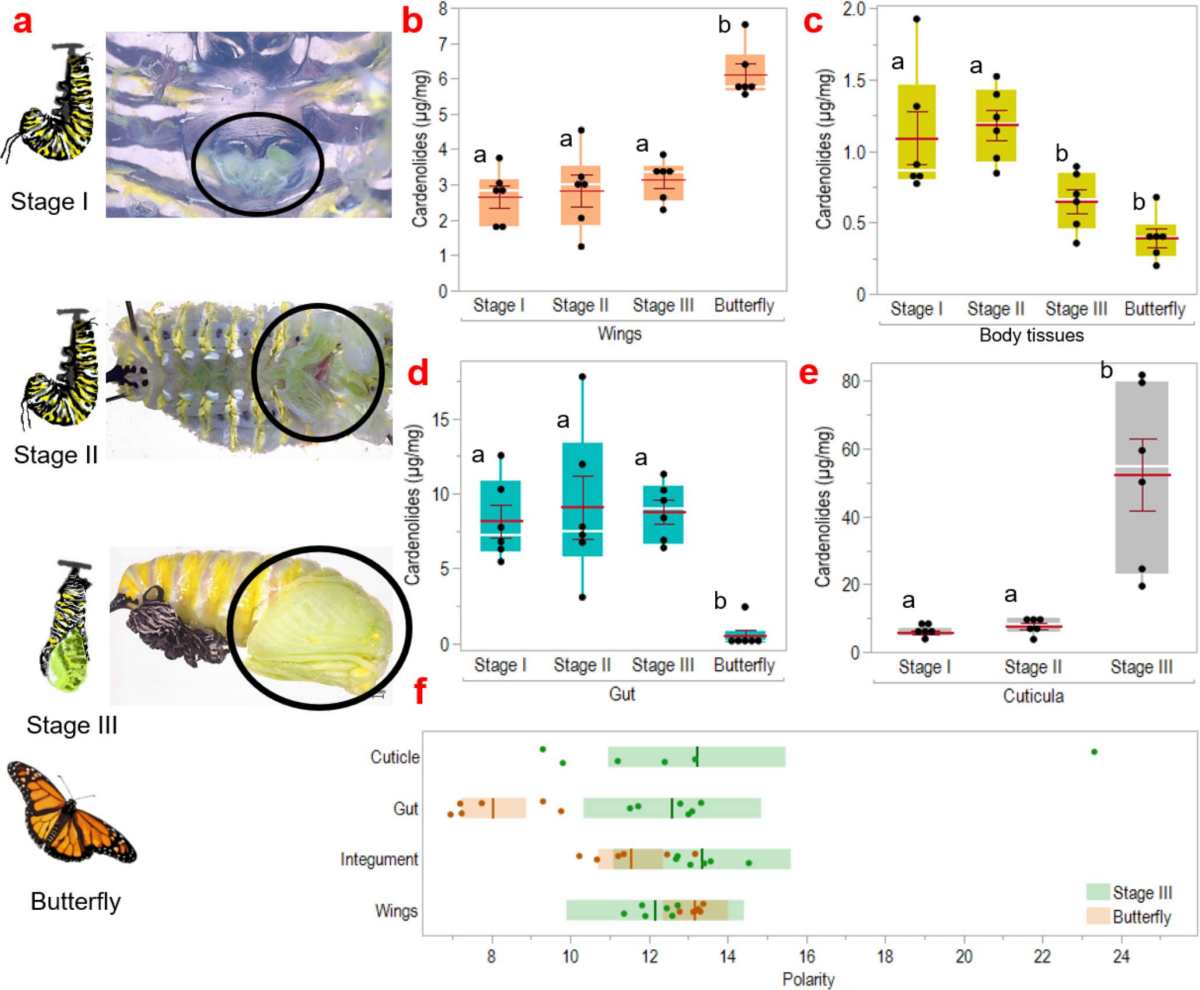
Cardenolide relocation from the pharate caterpillar to the adult stage in *D. plexippus.* We dissected L5 caterpillars that had just attached themselves for pupation (the so-called “J-hang” position) during three stages, up to the point when they began to shed their exuviae for pupation. These stages were categorized as follows: I = right after attachment, II = droopy tentacles, III = shedding the last caterpillar exuviae, and newly emerged butterfly, circles in images indicate wing discs (I, II) and pupal wings (III) (**a**). Cardenolide concentration in stages I-III and adult butterflies was measured in the wings (**b**), body tissues (**c**), and in the gut tissue (with content) (**d**). Cardenolides in cuticle were quantified in stages I-III (**e**). Boxes indicate interquartile ranges and white lines show the median. Red lines represent means, error bars indicate SE and black dots are individual data points. Different letters indicate significant differences between treatments. In addition, the polarity index of cardenolides found in gut, body tissue, wing and cuticle samples was calculated for stage III and adult butterflies (no cuticle samples). Each dot represents one sample, the lines show mean values and bars show confidence intervals (**f**). Adult butterflies did not contain cardenolides less polar than calactin (RT < 16 min)

**Fig. 4:**
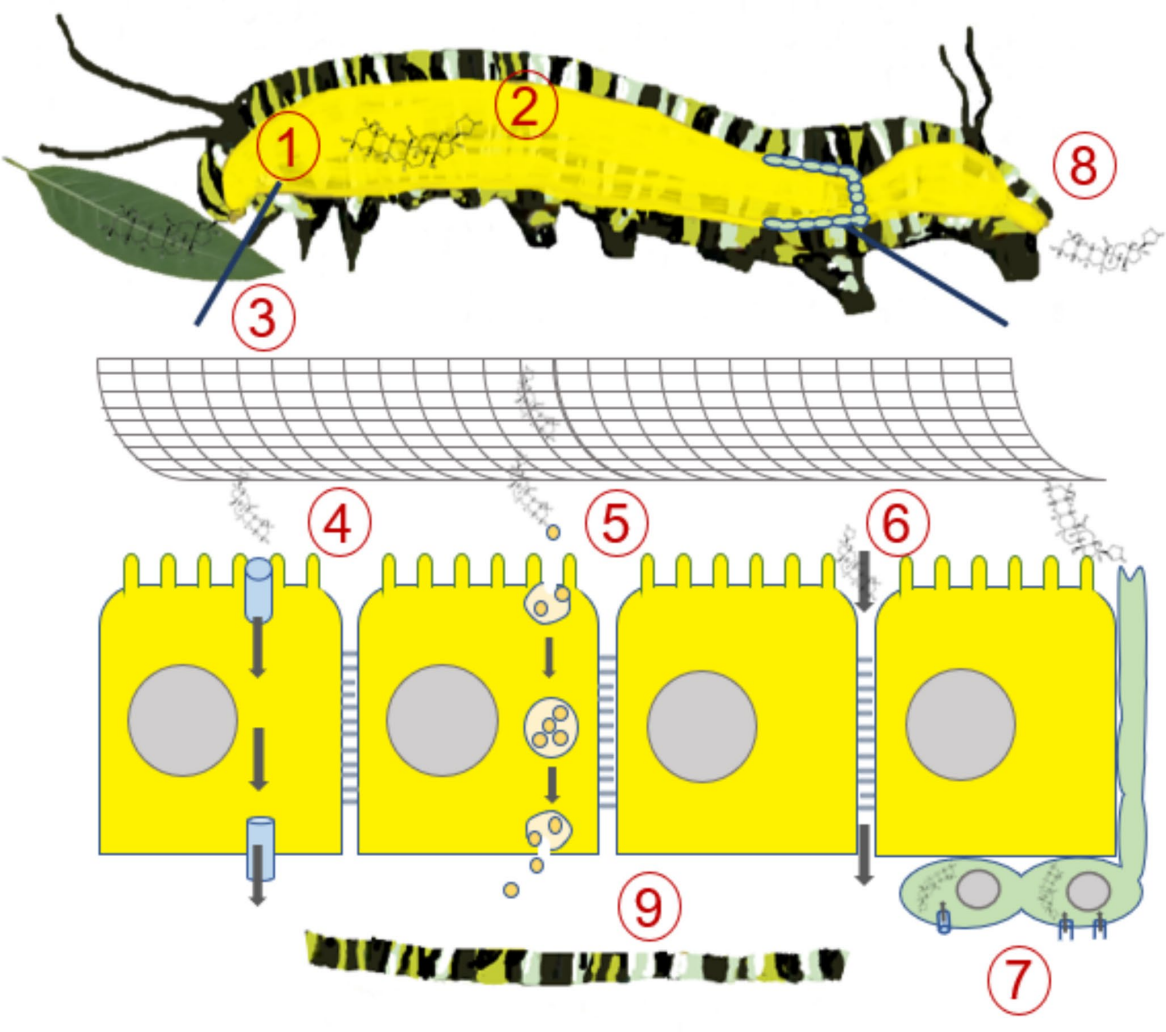
Synthesis of the possible fate of cardenolides in an insect after ingestion. During feeding, insects ingest cardenolides with plant material (**1**). In the midgut, cardenolides may be metabolized (**2**) (Seiber et al. 1980; Scudder and Meredith 1982; Marty and Krieger 1984; Jones et al. 2019; Agrawal et al. 2021) before crossing the peritrophic membrane, which may act as a first barrier (**3**) (Scudder and Meredith 1982; Barbehenn 1999). The toxins can either pass through the gut or may be retained and accumulate in the gut lumen via an unknown mechanism as suggested for monarch caterpillars (Petschenka and Agrawal 2015; Dreisbach et al. 2023). The gut epithelium is the second barrier that cardenolides must cross (Scudder and Meredith 1982; Dreisbach et al. 2023). Transcellular absorption (i.e. through the cells) of cardenolides via the gut epithelium may occur via active carrier-mediated processes (Detzel and Wink 1995; Frick and Wink 1995) or passively via diffusion (Yoder et al. 1976; Duffey et al. 1978; Scudder and Meredith 1982), depending on their chemical properties (**4**) (Agrawal et al. 2012). Another potential transcellular pathway is through endo- and exocytosis (**5**) (Dreisbach et al. 2023) although experimental evidence for this route is lacking. Alternatively, cardenolides could cross the gut epithelium via the paracellular route, i.e. through the septate junctions between the cells, which has been shown for certain proteins (**6**) (Casartelli et al. 2007; Huang et al. 2015). After passing through the gut epithelium, cardenolides may be detoxified in the hemocoel by binding to proteins (Agrawal et al. 2012) or by enzymatic alteration in the fat body (Marty and Krieger 1984). A role for detoxification of cardenolides was suggested for OATPs (Groen et al. 2017). Alternatively, they may be absorbed into the Malpighian tubules (**7**) (Torrie et al. 2004) and subsequently excreted (**8**). In glucosinolate sequestering leaf beetles it has been shown that specific transporters can prevent excretion by the Malpighian tubules (Yang et al. 2021). Similarly, the Malpighian tubules of *O. fasciatus* were shown to reabsorb ouabain against a strong concentration gradient (Meredith et al. 1984) and a comparable mechanism could explain the extended presence of ouabain in the hemolymph of monarch caterpillars compared to the other species observed here. In cardenolide sequestering insects, the toxins must remain functional and reach a storage compartment such as the integument in monarch caterpillars (Dreisbach et al. 2023) or the dorsolateral space in milkweed bugs (Duffey et al. 1978) (**9**), to mediate defense against predators (Duffey 1980; Despres et al. 2007; Beran and Petschenka 2022)

In addition to uptake studies focusing on the isolated midgut epithelium and the whole caterpillar, we examined the distribution and translocation of cardenolides across tissues from pupae to adult butterflies. In contrast to previous work by Nishio (1980), who conducted similar studies on *Asclepias humistrata* reared monarchs, we analyzed cardenolide levels across three stages from attachment of the larvae to chrysalis formation and immediately after hatching of the butterflies. In particular, we tested whether cardenolide concentrations and polarity patterns would change in wings, body tissues, gut, and cuticle during pupal development and in newly hatched butterflies. When we dissected caterpillars that had just attached themselves for pupation (J-hang position, stage I), imaginal discs had already begun to develop into wings. These small wings had increased in size in stages II (few hours after stage I) and III (around one hour after stage II; Fig. 3a). While cardenolides were already present in the developing wings, the concentration was much higher in adult wings (χ^2^_3_ = 71.36, *P* < 0.001; stage I– III vs. butterfly, *P* < 0.001 each, Fig. 3b). In the body tissues (excluding wings) cardenolide concentrations decreased until metamorphosis was complete (χ^2^_3_ = 29.29, *P* < 0.001; stage I– II vs. butterfly, *P* < 0.001 each, stage III vs. butterfly *P* = 0.43, Fig. 3c). Also, less cardenolides were found in the butterfly gut compared to the larval gut (χ^2^_3_ = 31.24, *P* < 0.001; stage I– III vs. butterfly, *P* < 0.001 each, Fig. 3d). In addition, caterpillars accumulated cardenolides in their exoskeleton during chrysalis formation (F_2,15_ = 17.82, *p* < 0.001; stage I–II vs. III, *P* < 0.001 each; Fig. 4e), which were lost when the caterpillars shed their last exuviae. Furthermore, the composition of cardenolides with respect to their polarity changed during metamorphosis in the gut (including contents), body tissues, and wings (Fig. 4f). The cardenolides removed with the last exuviae contained all cardenolides with a polarity index > 14, which are therefore no longer present in the adult butterfly. In the gut, the polarity of the cardenolides changed from medium polarity in stage III to more polar cardenolides in the butterfly after metamorphosis.

## Discussion

To date, there have been several studies on the possible fate of cardenolides during sequestration, leading to different hypotheses on how cardenolides are transported and stored in the insect.

### Uptake via the Gut (in Vitro)

Frick and Wink (1995) and Detzel and Wink (1995) studied the in vitro uptake of cardenolides using pieces of midgut epithelium of *O. fasciatus* (Detzel and Wink 1995) and *D. plexippus* (Frick and Wink 1995). Based on their studies, it was concluded that the uptake of digitoxin (Detzel and Wink 1995) or ouabain (Frick and Wink 1995) in the midgut epithelium occurs against a concentration gradient (Detzel and Wink 1995). In both model organisms, the accumulation of either ouabain (*D. plexippus*) or digitoxin (*O. fasciatus*) was found to be inhibited by the polar cardenolide convallatoxin, while digitoxin and ouabain did not compete as a substrate (Detzel and Wink 1995; Frick and Wink 1995). Furthermore, the uptake displayed an activation energy > 20 KJ/mol. Together, these findings indicated a carrier-mediated process, although an additional diffusion component was suggested (Detzel and Wink 1995; Frick and Wink 1995). We note, however, that immersion assays based on tissue pieces may be confounded by the lack of a pH-gradient and altered tissue polarity, which would be maintained in isolated and perfused guts. Organic Anion Transporting Polypeptides (OATPs) have been shown to facilitate the transport of ouabain across cell membranes in humans (Bossuyt et al. 1996; Kullak-Ublick et al. 2001), rats (Cattori et al., 2001), and fruit flies (Torrie et al. 2004). This has led to the hypothesis that OATPs might serve as potential cardenolide transporters in sequestering insects. To investigate this possibility, Baum (2015) conducted in vitro studies to assess the role of OATPs in ouabain transport in the sequestering leaf beetle *Chrysochus auratus* and its non-sequestering sister species *C. asclepiadeus*. The study, however, revealed that OATPs were unable to transport ouabain in either species. As a result, the authors concluded that OATPs are unlikely to be involved in cardenolide transport in *Chrysochus* species (Baum 2015).

In our transport studies, we found uptake of ouabain and uzarin across isolated perfused midguts of monarch caterpillars, although uzarin has been shown not be sequestered by monarch caterpillars in earlier studies (Dreisbach et al. 2023). This discrepancy may be due to the lack of the peritrophic membrane in our in vitro assay or other components of the complex physicochemical environment in the gut of living caterpillars. Remarkably, we found that also the midguts of the non-sequestering caterpillars of *E. core* and even *M. sexta*, which do not feed on cardenolide-containing plants, were permeable to ouabain. Nevertheless, the uptake was more efficient (i.e. occurred at a higher rate) in monarch caterpillars compared to the other species. Although our results do not allow us to infer the mechanisms of uptake, they provide new insights into how quantitative differences between species are mediated. In particular, they suggest that the capacity for sequestration in caterpillars per se is not (or at most quantitatively) mediated by the permeability of the gut epithelium.

### Uptake via the Gut (in Vivo)

Consistent with the in vitro results, we observed that, besides *D. plexippus*, the two non-sequestering species also absorb ouabain in vivo. The concentration of ouabain was particularly high in the hemolymph of *D. plexippus* after 5 h but decreased substantially after 24 h to a level similar to that of the other species. Surprisingly, ouabain concentrations in body tissues showed a similar pattern of decrease over time in all species and similar overall concentrations. Previous studies in *Oncopeltus fasciatus* by Scudder and Meredith (1982) in vivo, and Yoder et al. (1976) in vitro, showed that ouabain and digitoxin uptake was linearly correlated with concentration, was not saturable, and cardenolides did not accumulate against a concentration gradient, suggesting passive uptake. As noted above, other studies have suggested carrier-mediated transport in monarchs and *O. fasciatus* (Detzel, Frick). The presence of ouabain in the tissues of both *E. core* and *M. sexta* observed here could be due to either uptake via unspecific diffusion or a carrier-mediated mechanism not restricted to monarch caterpillars. However, we speculate that the presence of a specialized carrier for cardenolides in non-sequestering (*E. core*) or even non-cardenolide-feeding (*M. sexta*) lepidopteran species is rather unlikely. Rather, our data suggest that cardenolide sequestration may be a ubiquitous trait of lepidopteran guts, enhanced in the gut of sequestering danaine butterflies such as *D. plexippus*. Figure 4 provides an overview and summarizes the different mechanisms that have been proposed for sequestering and non-sequestering insects after cardenolide ingestion.

Although ouabain concentrations in the gut epithelium and body tissues of *D. plexippus* showed similar overall concentrations and a temporal decrease as in the other two species, we found higher concentrations in the hemolymph at 5 and 24 h compared to concentrations immediately after 90 min of ouabain feeding. This finding is consistent with our data on a higher sequestration rate in isolated guts. Why the higher concentrations in the hemolymph were not reflected by higher concentrations in the other tissues is an open question, but may be due to the fact that *D. plexippus* caterpillars were reared on *A. curassavica* causing a saturation of the cardenolide storage capacity. However, our data indicate that *D. plexippus* retains ouabain in the hemolymph longer than *E. core* and *M. sexta*, suggesting that reduced excretion of sequestered toxins, as described for glucosinolates in the horseradish flea beetle (Yang et al. 2021), may be a quantitative modulator of sequestration. Alternatively, the decrease in ouabain could be explained by conversion to unknown metabolites at different rates in the three species. The latter interpretation seems unlikely, however, since ouabain has been shown not to be metabolized by insects and other animals (Scudder and Meredith 1982; Kitano et al. 1998; Torrie et al. 2004). Similarly, the decrease in the gut epithelium samples and the body tissues of all species could be due to either excretion, degradation, or, in the case of the gut epithelia, transport into the hemocoel.

Our data suggest that lepidopteran gut epithelia may be generally permeable to ouabain, a feature that appears to vary among insect taxa. Barbehenn (1999) showed that ouabain permeates through the midgut epithelia of the generalist grasshopper *Melanoplus sanguinipes* (Orthoptera), whereas ouabain was not detected in the hemolymph of *Schistocerca gregaria* (Orthoptera) and *Periplaneta americana* (Blattodea) after oral feeding or intestinal administration. Notably, Barbehenn (1999) pointed out that the permeability of *M. sanguinipes* guts is even similar to that of the highly permeable compound gallic acid. Overall, the comparison of sequestration assays involving intact animals and based on isolated midguts may be confounded by efficient excretion from the hemocoel (Rafaeli-Bernstein and Mordue 1978) or by enzymatic degradation of cardenolides in the gut as it has been suggested to occur in caterpillars of *E. core* (Dreisbach et al. 2023). A further issue impairing the comparability of in vitro and in vivo assays is the great physicochemical complexity of the insect gut lumen. Allelochemicals that are lipophilic or amphiphilic are anticipated to be stored within lipid aggregates, known as micelles, in the fluid phase of the gut in herbivorous insects, corresponding to their degree of lipophilicity (Barbehenn 1999). In other words, the gut contents are likely to interfere with sequestration in whole caterpillars compared to assays involving isolated gut tissues. Putative micelles or segregation of milkweed cardenolides has also been suggested to occur in the gut lumen of monarch caterpillars (Dreisbach et al. 2023).

### Time Dependent Cardenolide Sequestration in *D. plexippus*

When we investigated digitoxin and ouabain uptake in vivo in a time-dependent manner, we found that digitoxin uptake was more efficient, which is in line with previous studies that suggested a 14-fold faster uptake of digitoxin than ouabain in *O. fasciatus* (Scudder and Meredith 1982). When we examined the time-dependent uptake of natural cardenolides from the host plant *A. curassavica*, we found that the epithelial tissue contained only relatively apolar cardenolides at different time samples (and in particular, one at RT 18.6 min). Therefore, highly apolar cardenolides are likely retained longer in the midgut or metabolized to more polar forms, since we observed a simultaneous accumulation of medium-polar cardenolides in the body tissues but did not find the cardenolide mentioned above. Our results further show that sequestration in *D. plexippus* caterpillars is a very rapid process that occurs as soon as cardenolides reach the midgut. Remarkably, we found that medium polar cardenolides such as calotropin and also a more lipophilic cardenolide (RT 20.2 min) were present in the body tissues already 1 min after ingestion of *A. curassavica* leaves.

### Importance of Cardenolide Polarity

The polarity of the cardenolide determines the rate of sequestration, and cardenolides that are less polar than calactin are likely to be metabolized to intermediate polar cardenolides (Seiber et al. 1980; Marty and Krieger 1984). For example, in *D. plexippus* it has been demonstrated that the apolar uscharidin is metabolized in the gut tissue to the more polar calotropin and calactin (configurational isomers) (Marty and Krieger 1984; Seiber et al. 1980). Also, in *O. fasciatus* it was shown that in the Malpighian tubules and possibly dorsolateral space epithelia, the order of glycoside permeability was reversed compared to the gut (polar favored over apolar) (Duffey et al. 1978; Scudder and Meredith 1982). In addition, the polarity of the sequestered cardenolides appears to play an important role in storage. This is probably the reason why *D. plexippus* raised on *A. curassavica* mainly sequesters the isomers calotropin and calactin, which, due to their polarity, may be difficult to mobilize in the hemolymph and therefore potentially easier to store (Rosenthal and Berenbaum 1992).

### Cardenolide Translocation During Metamorphosis

We speculate that lipophilic cardenolides in particular, but also other cardenolides stored in the cuticle, cannot be mobilized and are removed by the final exuviae during pupation and are therefore not present in the butterfly. We found that shedding the last exuviae corresponds to a total loss of 163–540 µg of cardenolides. This is consistent with studies by Nishio (1980), who found a 25% loss of cardenolides with shedding the last exuviae in monarchs reared on *A. humistrata*. Furthermore, we detected increasing concentrations of cardenolides in the developing wings while concentrations in the gut decreased during metamorphosis, suggesting relocation of sequestered toxins.

Previously, it was proposed that after pupation, the gut fluid rich in polar cardenolides can pass through the gut membrane into the hemolymph pool under the wings (Nishio 1980). We found, that this reddish gut fluid is already present in stage I (see supplementary figure S3). Therefore, we speculate that it may develop along with the development of the imaginal discs into wings. Nishio et al. (1980) observed that the reddish fluid in the butterfly gut shortly before hatching is present only in the hindgut, whereas at hatching the fluid throughout the entire gut is green in color. We made the same observation, suggesting that the red fluid in the gut is completely absorbed during metamorphosis. In addition, we found that the concentration of cardenolides in the wings increased twofold, while it decreased in the gut fluid and body tissues of adult butterflies, leading us to speculate that cardenolides in the hemolymph are sequestered in the soft wing scales during wing inflation.

In summary, our data suggest that the permeability for cardenolides per se is not limited to the midgut epithelium of *D. plexippus* but is also found in the gut of other caterpillars, including species that are not closely related. This observation makes the involvement of a specific carrier mechanism rather unlikely. While the underlying transport mechanisms are still unclear, the uptake of polar ouabain and unpolar digitoxin across the monarch gut appears to be more efficient than in other species, and cardenolides may be retained longer in the monarch hemolymph, suggesting that a combination of gut permeability and excretion may mediate the quantitative differences in sequestration observed between species. In addition, sequestration is remarkably rapid, and cardenolides have been detected in body tissues as early as 1 min after feeding milkweed leaves. Large amounts of cardenolides stored in the cuticle are lost during metamorphosis. Adult butterflies contain no less polar cardenolides than calactin, with the largest amounts found in butterfly wings, probably derived from cardenolides accumulated in the gut fluid during metamorphosis.

## Electronic Supplementary Material

Below is the link to the electronic supplementary material.

Supplementary Material 1

Supplementary Material 2

## Data Availability

Data is provided within the supplementary information files.

## References

[CR1] Agrawal AA, Böröczky K, Haribal M, Hastings AP, White RA, Jiang R-W, Duplais C (2021) Cardenolides, toxicity, and the costs of sequestration in the coevolutionary interaction between monarchs and milkweeds. Proceedings of the National Academy of Sciences 118(16):e2024463118 10.1073/pnas.2024463118 PMC8072370 33850021

[CR2] Agrawal AA, Petschenka G, Bingham RA, Weber MG, Rasmann S (2012) Toxic cardenolides: chemical ecology and coevolution of specialized plant–herbivore interactions. New Phytol 194(1):28–45 22292897 10.1111/j.1469-8137.2011.04049.x

[CR3] Barbehenn RV (1999) Non-absorption of ingested lipophilic and amphiphilic allelochemicals by generalist grasshoppers: the role of extractive ultrafiltration by the peritrophic envelope. Archives Insect Biochem Physiology: Published Collab Entomol Soc Am 42(2):130–137 10.1002/(SICI)1520-6327(199910)42:2<130::AID-ARCH3>3.0.CO;2-C 10504206

[CR4] Baum M (2015) Transmembrane carriers of cardenolide-adapted leaf beetles (Coleoptera, Chrysomelidae). Staats-und Universitätsbibliothek Hamburg Carl von Ossietzky

[CR5] Beran F, Petschenka G (2022) Sequestration of plant defense compounds by insects: from mechanisms to insect–plant coevolution. Ann Rev Entomol 67:163–180 34995091 10.1146/annurev-ento-062821-062319

[CR7] Bossuyt X, Müller M, Meier PJ (1996) Multispecific amphipathic substrate transport by an organic anion transporter of human liver. J Hepatol 25(5):733–738 8938553 10.1016/s0168-8278(96)80246-7

[CR6] Brower LP, Moffitt CM (1974) Palatability dynamics of cardenolides in the monarch butterfly. Nature 249(5454):280–283 4833249 10.1038/249280b0

[CR8] Casartelli M, Corti P, Cermenati G, Grimaldi A, Fiandra L, Santo N, Pennacchio F, Giordana B (2007) Absorption of horseradish peroxidase in *Bombyx mori* larval midgut. J Insect Physiol 53(6):517–525 17391693 10.1016/j.jinsphys.2007.02.004

[CR9] CaTtori V, Montfoort JE, Stieger B, Landmann L, Meijer DK, Winterhalter KH, Meier PJ, Hagenbuch B (2001) Localization of organic anion transporting polypeptide 4 (Oatp4) in rat liver and comparison of its substrate specificity with Oatp1, Oatp2 and Oatp3. Pflügers Archiv 443:188–195 11713643 10.1007/s004240100697

[CR10] Despres L, David J-P, Gallet C (2007) The evolutionary ecology of insect resistance to plant chemicals. Trends Ecol Evol 22(6):298–307 17324485 10.1016/j.tree.2007.02.010

[CR11] Detzel A, Wink M (1995) Evidence for a cardenolide carrier in *Oncopeltus fasciatus* (Dallas) (Insecta: Hemiptera). Z für Naturforschung C 50(1–2):127–134

[CR12] Dobler S, Petschenka G, Wagschal V, Flacht L (2015) Convergent adaptive evolution–how insects master the challenge of cardiac glycoside-containing host plants. Entomol Exp Appl 157(1):30–39

[CR13] Dow JAT (1981) Countercurrent flows, water movements and nutrient absorption in the Locust midgut. J Insect Physiol 27(9):579–585

[CR14] Dreisbach D, Bhandari DR, Betz A, Tenbusch L, Vilcinskas A, Spengler B, Petschenka G (2023) Spatial metabolomics reveal divergent cardenolide processing in the monarch (*Danaus plexippus*) and the common crow butterfly (*Euploea core*). Mol Ecol Resour 23(6):1195–1210 36941779 10.1111/1755-0998.13786

[CR15] Duffey SS (1980) Sequestration of plant natural products by insects. Ann Rev Entomol 25(1):447–477

[CR16] Duffey SS, Blum MS, Isman MB, Scudder GGE (1978) Cardiac glycosides: a physical system for their sequestration by the milkweed bug. J Insect Physiol 24(8–9):639–645

[CR17] Fiandra L, Casartelli M, Cermenati G, Burlini N, Giordana B (2009) The intestinal barrier in lepidopteran larvae: permeability of the peritrophic membrane and of the midgut epithelium to two biologically active peptides. J Insect Physiol 55(1):10–18 18948109 10.1016/j.jinsphys.2008.09.005

[CR18] Frick C, Wink M (1995) Uptake and sequestration of ouabain and other cardiac glycosides in *Danaus plexippus* (Lepidoptera: Danaidae): evidence for a carrier-mediated process. J Chem Ecol 21(5):557–575 24234250 10.1007/BF02033701

[CR19] Groen SC, LaPlante ER, Alexandre NM, Agrawal AA, Dobler S, Whiteman NK (2017) Multidrug transporters and organic anion transporting polypeptides protect insects against the toxic effects of cardenolides. Insect Biochem Mol Biol 81:51–61 28011348 10.1016/j.ibmb.2016.12.008 PMC5428987

[CR21] Holzinger F, Frick C, Wink M (1992) Molecular basis for the insensitivity of the Monarch (*Danaus plexippus*) to cardiac glycosides. FEBS Lett 314(3):477–480 1334851 10.1016/0014-5793(92)81530-y

[CR22] Holzinger F, Wink M (1996) Mediation of cardiac glycoside insensitivity in the monarch butterfly (*Danaus plexippus*): role of an amino acid substitution in the ouabain binding site of Na+, K+-ATPase. J Chem Ecol 22(10):1921–1937 24227116 10.1007/BF02028512

[CR23] Huang J-H, Jing X, Douglas AE (2015) The multi-tasking gut epithelium of insects. Insect Biochem Mol Biol 67:15–20 25982023 10.1016/j.ibmb.2015.05.004 PMC4644519

[CR24] Jones PL, Petschenka G, Flacht L, Agrawal AA (2019) Cardenolide intake, sequestration, and excretion by the monarch butterfly along gradients of plant toxicity and larval ontogeny. J Chem Ecol 45(3):264–277 30793231 10.1007/s10886-019-01055-7

[CR25] Kitano S, Morimoto S, Nishibe A, Fukuo K, Hirotani A, Nakahashi T, Yasuda O, Ogihara T (1998) Exogenous ouabain is accumulated in the adrenals and mimics the kinetics of endogenous digitalis-like factor in rats. Hypertens Res 21(1):47–56 9582108 10.1291/hypres.21.47

[CR26] Kullak-Ublick GA, Ismair MG, Stieger B, Landmann L, Huber R, Pizzagalli F, Fattinger K, Meier PJ, Hagenbuch B (2001) Organic anion-transporting polypeptide B (OATP-B) and its functional comparison with three other OATPs of human liver. Gastroenterology 120(2):525–533 11159893 10.1053/gast.2001.21176

[CR27] Kumar P, Pandit SS, Steppuhn A, Baldwin IT (2014) Natural history-driven, plant-mediated RNAi-based study reveals CYP6B46’s role in a nicotine-mediated antipredator herbivore defense. Proceedings of the National Academy of Sciences 111(4):1245–1252 10.1073/pnas.1314848111 PMC3910579 24379363

[CR29] Malcolm SB, Zalucki (eds) (1996) Milkweed latex and cardenolide induction may resolve the lethal plant defence paradox. In Proceedings of the 9th international symposium on insect-plant relationships, Springer Netherlands pp. 193–196

[CR28] Malcolm S, Rothschild M (1983) A danaid mullerian mimic, Euploea core amymone (Cramer) lacking cardenolides in the pupal and adult stages. Biol J Linn Soc 19(1):27–33

[CR30] Martin RA, Lynch SP, Brower LP, Malcolm SB, van Hook T (1992) Cardenolide content, emetic potency, and thin-layer chromatography profiles of monarch butterflies, *Danaus plexippus*, and their larval host-plant milkweed, *Asclepias humistrata*, in Florida. Chemoecology 3(1):1–13

[CR31] Marty MA, Krieger RI (1984) Metabolism of uscharidin, a milkweed cardenolide, by tissue homogenates of monarch butterfly larvae, Danaus plexippus L. J Chem Ecol 10:945–956 24318786 10.1007/BF00987975

[CR32] Meredith J, Moore L, Scudder GG (1984) Excretion of ouabain by malpighian tubules of *Oncopeltus fasciatus*. Am J Physiology-Regulatory Integr Comp Physiol 246(5):R705–R715 10.1152/ajpregu.1984.246.5.R705 6720994

[CR33] Nishida R (2002) Sequestration of defensive substances from plants by Lepidoptera. Ann Rev Entomol 47(1):57–92 11729069 10.1146/annurev.ento.47.091201.145121

[CR34] Nishio S (1980) The fates and adaptive significance of cardenolides sequestered by larvae of *Danaus plexippus* (L.) and *Cycnia inopinatus* (Hy. Edwards). Dissertation, University of Georgia

[CR35] Opitz SEW, Müller C (2009) Plant chemistry and insect sequestration. Chemoecology 19(3):117–154

[CR36] Petschenka G, Agrawal AA (2015) Milkweed butterfly resistance to plant toxins is linked to sequestration, not coping with a toxic diet. Proc Royal Soc B: Biol Sci 282(1818):20151865 10.1098/rspb.2015.1865 PMC4650158 26538594

[CR37] Petschenka G, Pick C, Wagschal V, Dobler S (2013) Functional evidence for physiological mechanisms to circumvent neurotoxicity of cardenolides in an adapted and a non-adapted hawk-moth species. Proc Royal Soc B: Biol Sci 280(1759):20123089 10.1098/rspb.2012.3089 PMC3619502 23516239

[CR20] Heckel DG (2014) Insect detoxification and sequestration strategies. Ann Plant Rev 47:77–114

[CR38] Rafaeli-Bernstein ADA, Mordue W (1978) The transport of the cardiac glycoside ouabain by the malpighian tubules of Zonocerus Variegatus. Physiol Entomol 3(1):59–63

[CR39] Rasmann S, Agrawal AA (2011) Latitudinal patterns in plant defense: evolution of cardenolides, their toxicity and induction following herbivory. Ecol Lett 14(5):476–483 21371232 10.1111/j.1461-0248.2011.01609.x

[CR40] Rosenthal GA, Berenbaum MR (1992) Herbivores: Their Interactions with Secondary Plant Metabolites: Volume II: Ecological and Evolutionary Processes. 01259718

[CR41] Rothschild ML, von Euw J, Reichstein T, Smith DAS, Pierre J (1975) Cardenolide storage in *Danaus chrysippus* (L.) with additional notes on *D. plexippus* (L.). Proceedings of the Royal Society of London. Series B. Biological Sciences 190 (1098):1–31

[CR42] Salisbury L, Salisbury C (2000) A monarch diary. Blue Jay 58 (2)

[CR43] Scudder GGE, Meredith J (1982) The permeability of the midgut of three insects to cardiac glycosides. J Insect Physiol 28(8):689–694

[CR44] Seiber JN, Tuskes PM, Brower LP, Nelson CJ (1980) Pharmacodynamics of some individual milkweed cardenolides fed to larvae of the monarch butterfly (*Danaus plexippus* L). J Chem Ecol 6(2):321–339

[CR45] Torrie LS, Radford JC, Southall TD, Kean L, Dinsmore AJ, Davies SA, Dow JAT (2004) Resolution of the insect ouabain paradox. Proc Natl Acad Sci 101(37):13689–13693 15347816 10.1073/pnas.0403087101 PMC518814

[CR46] Vaughan GL, Jungreis AM (1977) Insensitivity of lepidopteran tissues to ouabain: physiological mechanisms for protection from cardiac glycosides. J Insect Physiol 23(5):585–589

[CR47] Wolfersberger MG, Spaeth DD, Dow JAT (eds) (1986) Permeability of the peritrophic membrane of tobacco hornworm larval midgut. American Zoologist, New Hampshire

[CR48] Yang Z-L, Nour-Eldin HH, Hänniger S, Reichelt M, Crocoll C, Seitz F, Vogel H, Beran F (2021) Sugar transporters enable a leaf beetle to accumulate plant defense compounds. Nat Commun 12(1):2658 33976202 10.1038/s41467-021-22982-8 PMC8113468

[CR49] Yoder CA, Leonard DE, Lerner J (1976) Intestinal uptake of ouabain and digitoxin in the milkweed bug, *Oncopeltus fasciatus*. Experientia 32(12):1549–1550. 10.1007/BF01924445

