# Supplementary material for: Regionality and Temporal Dynamics of Sequestration and Relocation of Cardenolides in the Monarch Butterfly, Danaus plexippus
Source: J Chem Ecol. 2025 Feb 4;51(1):19. doi: 10.1007/s10886-025-01572-8 (PMC11794346; doi:10.1007/s10886-025-01572-8)
Supplement: Supplementary file 1 — Supplementary Material 1 [file 10886_2025_1572_MOESM1_ESM.docx]

# SUPPLEMENTARY MATERIAL

Regionality and Temporal dynamics of sequestration and Relocation of cardenolides in the monarch butterfly, *Danaus plexippus*

Authors: Anja Betz^1^***, Birgit Höglinger^1^, frank Walker^1^, Georg Petschenka^1^*

*Affiliation:*

*^1^Department of Applied Entomology, University of Hohenheim; Stuttgart, Germany*

**Corresponding authors. Emails:* [*Anja.betz@googlemail.com*](mailto:Anja.betz@googlemail.com), [*Georg.Petschenka@uni-hohenheim.de*](mailto:Georg.Petschenka@uni-hohenheim.de)

# Methods

*Ouabain and Digitoxin Sequestration.* To compare the sequestration of a relatively polar and a relatively unpolar cardenolide in whole monarch caterpillars, we used the non-milkweed cardenolides ouabain and digitoxin (Carl Roth GmbH, Karlsruhe, Germany) which were both shown to be sequestered by monarch caterpillars before. Ouabain is a polar cardenolide, and digitoxin is a apolar cardenolide. Both cardenolides were prepared at a concentration of 10^-2^ M in methanol. Next, we pipetted 20 µl of each toxin solution in circular movements onto both sides of leaf discs, punched from *A. curassavica* using a cork borer (20 mm ⌀). After evaporation of the methanol, L5 caterpillars raised on *A. curassavica* were placed in Petri dishes and fed with either digitoxin or ouabain treated leaf discs. Consumed leaf discs were replaced with fresh leaf discs treated in the same manner. At 5, 10, 30, 60, 90, 120, and 180 min after the start of feeding (note intermittent feeding), caterpillars were harvested for chemical analysis (n = 4, per time point and treatment). Caterpillars directly taken from *A. curassavica* that had not been exposed to ouabain and digitoxin were used as a control. At the respective timepoint, a hemolymph sample (10 µl) was collected as described above, and caterpillars were frozen at – 20°C for later dissection. For the analysis of digitoxin and ouabain, the gut epithelium (PM and dietary content removed) and the body tissues (i.e. the caterpillar integument plus the adhering tissues) were dissected and processed separately. Tissue samples were extracted as described below and digitoxin and ouabain were quantified by HPLC-MS.


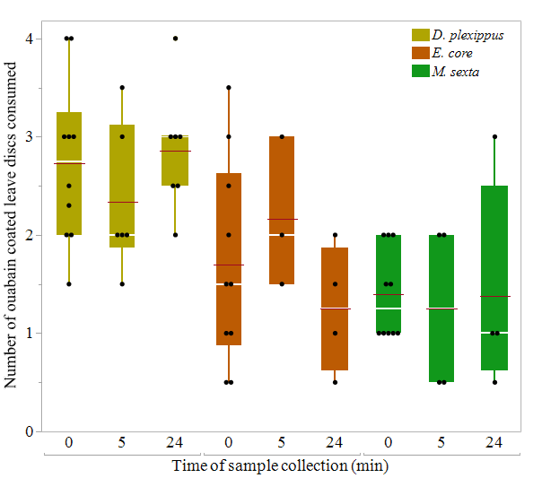


**S1.** *Number of ouabain-coated leaf discs consumed by species and time point.* Each caterpillar was offered ouabain-coated leaf discs ad libitum for 90 min. Consumed discs for this time period are shown, separated by later sample collection times. Zero indicates that the caterpillar was sampled immediately after 90 min. White lines indicate median, red lines indicate mean, and black dots visualize raw data points.


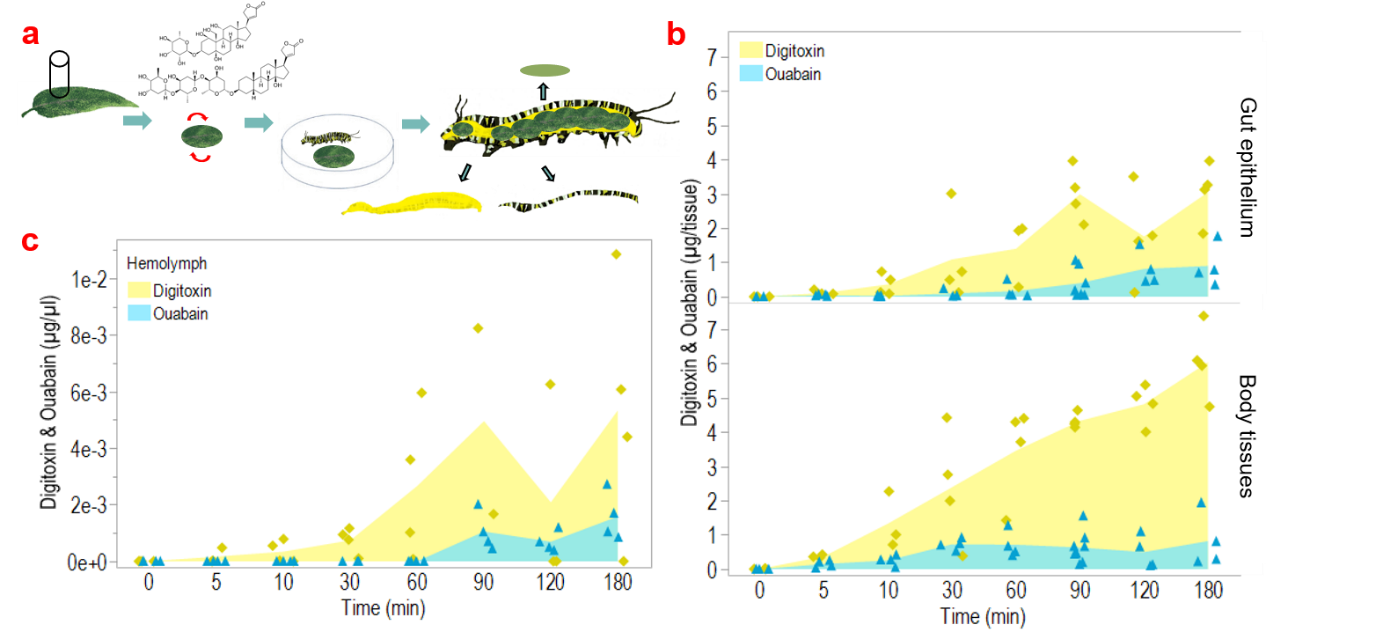


**S2.** S*equestration of ouabain and digitoxin in caterpillars of D. plexippus.* Caterpillars were fed with ouabain and digitoxin as illustrated in the experimental setup (a). Total amounts of ouabain and digitoxin were determined by HPLC-MS in dissected gut epithelia and body tissues (b) and in the hemolymph over time (c). Edges of colored areas connect the means of the raw data. Raw data are shown as yellow diamonds (digitoxin) and blue triangles (ouabain).


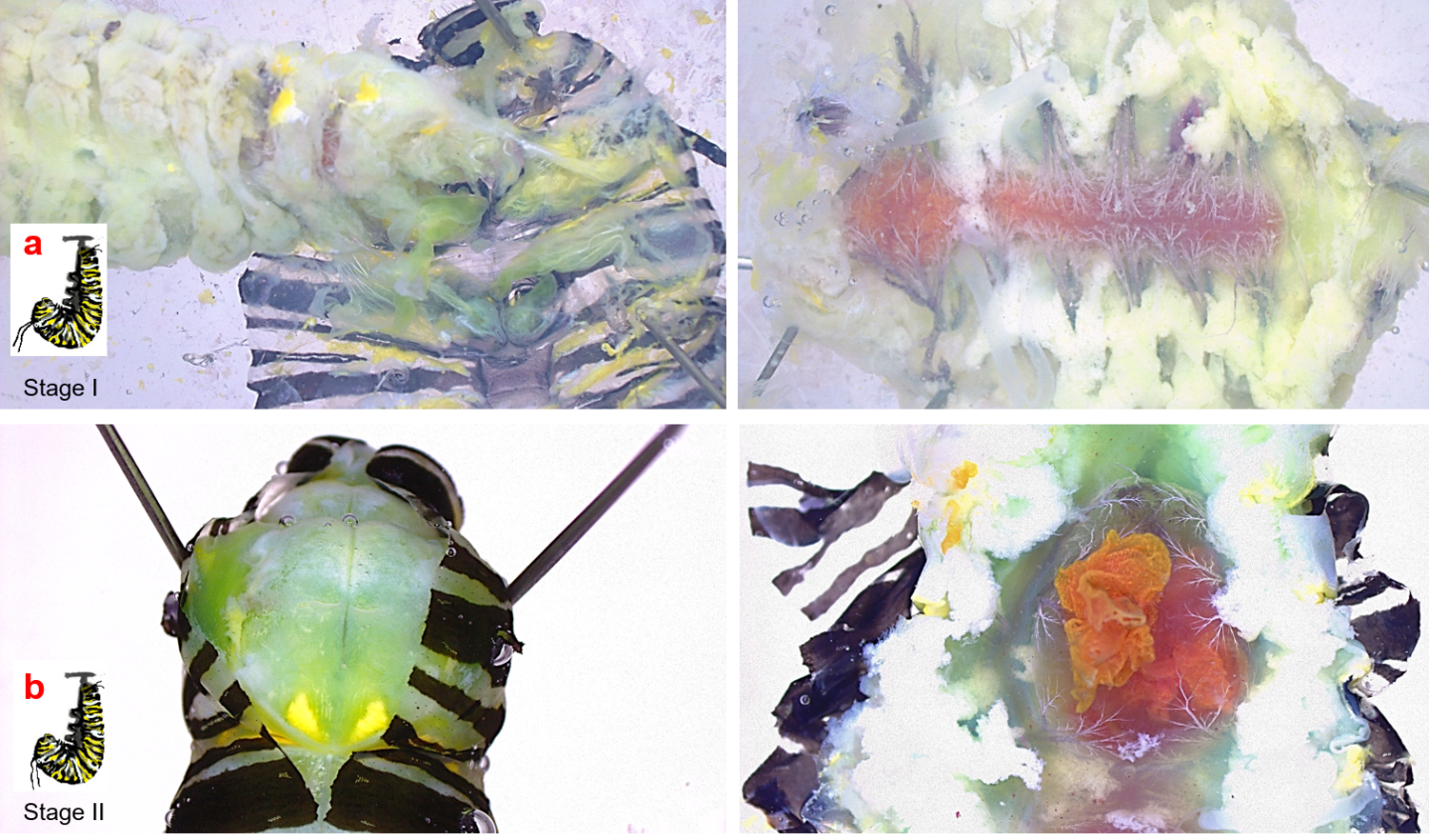


**S3***. Removal of cuticle during stages.* The pictures show the separation of the cuticle from the body tissues in stage I (a) and stage II (b). In stage III, the last exuviae are shed during pupation and can be easily collected. On the right hand side, corresponding images of the reddish colored gut fluid are shown according to the stage.
